# Navigating disruptive experiences in combat sports: perspectives of Brazilian, Portuguese, and Spanish masters on emotional control, resilience, and well-being

**DOI:** 10.3389/fspor.2025.1675930

**Published:** 2025-10-23

**Authors:** Maria Gabriela dos Santos, Yan Lazáro Santos, Thabata Castelo Branco Telles, Cristiano Roque Antunes Barreira

**Affiliations:** ^1^ Phenomenology and Body Practices Research Group (GFEPAC), Ribeirão Preto School of Physical Education and Sport, University of São Paulo, Ribeirão Preto, Brazil; ^2^ Phenomenology and Body Practices Research Group (GFEPAC), School of Philosophy, Sciences and Literature of Ribeirão Preto, Department of Psychology, University of São Paulo, Ribeirão Preto, Brazil; ^3^ University of Maia (UMAIA) and Politechnic Institute of Maia (IPMAIA), N2i, Braga, Portugal

**Keywords:** ethics, martial arts, sport psychology, teaching, violence

## Abstract

No other sport has such a fine line with violence as combat sports, but it is understood that in order to improve the practitioner there are psychological-combative transitions between the phenomena of playing, corporal fighting and brawling. This is not to omit the relevance of this proximity, but to try to understand it. So how can this experiential proximity between sport and violence lead to the development of the practitioner, from the masters’ perspective? This study adopted an empirical-phenomenological approach, in which interviews were conducted through a mode of suspensive listening. Following this, Psychological Reduction and Intentional Crossing were employed to analyze 47 interviews with teachers from all five regions of Brazil, the northern region of Portugal, and the northwestern and central-northern region of Spain. For now, across the three cultures investigated, we have identified and described four Units of Meaning that represent different facets of the manifestations of the phenomenon in question: 1. Combative Intensification; 2. Disruptive Situations; 3. Interventions by the instructor; 4. Changes in the Process. It is concluded that, from the teacher's perspective and through the comparison of three different countries, for the practitioner to develop, the combative situation must be ethically and pedagogically grounded in the necessary intensification that allows the learner to maintain the fighting spirit. However, when the teacher perceives that the student is not self-controlled in the moment, they intervene with the aim of promoting change and supporting the student's development in the practice of combat sports.

## 1 Introduction

The field of Martial Arts and Combat Sports (MA&CS) is a multifaceted phenomenon that gives rise to a continuous stream of discussion, including the experience of disruptive situations. It is pertinent to inquire whether practices of combat that encompass hitting and grabbing another individual, and being hit and grabbed, albeit deliberately and gradually, can genuinely facilitate personal development. The question thus arises as to how this may be achieved (1).

It can be argued that a violent phenomenon is such a disruptive^1^ experience that it crosses the boundary from a game, a fight or a sport, when viewed through the lens of Psychology of Martial Arts and Combat Sports (2). It is therefore anticipated that the practice of combative sports will entail a specific approach from the disciplines of psychology (3–10), pedagogy (5, 11–14) and ethics (6, 9, 15–17). The present investigation is grounded in the phenomenological model structured by Barreira et al. (1). Furthermore, although there are consolidated contributions to the development of fighting knowledge in MA&CS (18, 19), such proposals do not explicitly consider psychological transitions between playfulness and violence, which is a central focus in the present analysis.

If, on the one hand, Ciaccioni et al. (20) identified a broad thematic scope in their review on martial arts and mental health, on the other hand, a recurrent feature of combat practices is the presence of aggressiveness. Nevertheless, this issue of MA&CS can still be approached from a variety of perspectives (21, 22). Contemporary bibliographies reveal a divided scholarly landscape: on the one hand, some studies focus on the potential of combat practices to incite violence (17, 23, 24); on the other hand, others emphasize their capacity to foster personal development throughout the course of practice (4, 6, 17, 25).

Although the relationship may not be direct, the perception that the apprentice's behaviour is closely linked to the instructor's teachings in MA&CS practices is less controversial (9, 26, 27). In particular, the ethical dimension of these modalities is elucidated by Barreira [(28), p. 209–210]: “It is not feasible to quantify the full moral impact of the relationship between a master and a disciple. However, the role of the master in the martial arts context makes it clear that this impact cannot be ignored. It is during practice and training that appropriate and inappropriate behaviour is taught. Regardless of the means employed, it is evident that the ethical dimension of a martial art is shaped by the master's relationship with their students and disciples”.

In a similar perspective, Simões et al. [(17), p. 840] state: “With this work it is possible to conclude that the practice of MA&CS is an excellent means of biopsychosocial, ethical and aesthetic development for children and adolescents, as long as training is conducted by qualified professionals with impeccable ethics [..]”.

In addition to the recurring discourses in MA&CS that are uplifting in nature and the idealisation presumed by an “irreproachable ethic”, there is currently a paucity of psychological evidence that demonstrates how the practitioner's personal development can occur through the lived experience of the proximity between violence and corporal fighting in MA&CS. Consequently, it was only feasible to examine the intended outcomes by drawing upon specific research that sought to understand how practitioners experience these phenomena. This included the phenomenological approach that has been a foundational tenet for over a decade (8, 27–35).

By immersing oneself in the lived experience of MA&CS practitioners, one can gain insight into the nuances of ambivalence and ambiguity. To this end, it is essential to argue for the need to focus on each person's sensitive norm (30). For those who practise MA&CS, the phenomenon of violence is often not defined externally. This stands in contrast to the physicalist perspective, which defines violence in terms of pain and injury caused by blows, or the idealist perspective, which frames it as the objective violation of a rule. Instead, violence is constituted within the lived experience itself. By attending to this ambivalent and conceptually elusive territory, the experiences in question have been studied through the lens of psychological transitions (34).

As the authors themselves explain, the phenomenological discernment of combative actions associated with combat practices – which includes such activities as play-fighting, corporal fighting, brawling, duelling, self-defence and instrumental offensive combat [(34), p. 36] – does not neglect the close relationship between corporal fighting and the phenomenon of violence. This opens the way for the study of how violence is experienced and what consequences it may entail. While MA&CS practices may have imaginary or potential real-world implications in an institutional-educational context, they are primarily conducted as teaching, learning, training, and competition. Consequently, it is unlikely that these practices will unfold in the form of duels, self-defense, or instrumental offensive combat. Accordingly, when differentiating combat phenomena, psychological research tends to focus on the combative transitions between play-fighting, corporal fighting, and brawling.

Fighting can be defined as an action with physical, psychological, and spiritual qualities. It encompasses the willingness to dominate the opponent's bodily mobility through the use of techniques and gestures permitted within each modality. Furthermore, the motivation behind fighting is intrinsic rather than extrinsic. It is not driven by a desire to confront, to restore honour, or to engage in playful combat. Rather, the essence of its motivation can be described as “combat for combat's sake” [(29), p. 429]. In this way, it has been established that the recurring challenge for an MA&CS practitioner is to maintain the lived experience of combat as a fight – avoiding both the complacent experience of play and, even more so, the brute experience of a brawl.

Nevertheless, combat can resemble a violent conflict between individuals who engage in defensive and offensive actions, such as instrumental offensive combat, duelling, self-defence, and brawling. The continuation of the fight thus depends on the conquest of a subjective disposition that is constantly under threat—a disposition that resists collapsing either into a state of play, where complacent experience prevails and the gravity of combat is undermined by the absence of a will to win, or into the raw experience of a brawl, where anger and fear blind the subject, leading them to perceive the other as nothing but a thing and to attack indiscriminately. It is not necessary to exclude these experiences entirely from the experience of fighting, nor must they predominate to the point of determining the practitioner's actions. Such predominance would entail the collapse of the fight into either complacency or violence (34).

“Physical combat can be seen as one of the most concrete, extreme and elementary ways in which empathy, or the recognition of the other, can be developed or obscured in human relationships” [(36), p. 369]. This observation further reinforces the argument that there are conceptual inconsistencies within the field of MA&CS. In order to recognise actions between two practitioners who acknowledge each other as different selves, the first and most fundamental basis is empathy. In accordance with Edith Stein's phenomenological analyses (37), this is the necessary condition for understanding otherness during a fighting experience. However, this condition of recognising the other can change, potentially leading to an erasure of the self. This tension can lead to the phenomena of combative psychological transition, making the challenge of sustaining the fight appear as the most essential for the psychological understanding of the martial artist's eventual maturation. The question thus arises as to how experiencing these transitions may affect the practitioner's behaviour.

It can be seen that the ability to act in a given situation is not static but rather depends on a number of factors. These include the actions of the participants themselves, the norms that they adhere to, their awareness of the sensitivity of others and the way in which the situation is handled. This aspect has an impact on the principles of rule and the degree of unpredictability that can be expected in a given situation. It thus falls upon the instructor to guarantee the appropriate supervision of the combat environment in each instance, thereby creating an environment that encourages the acceptance of unpredictability as a manageable risk. The determination to fight, that is, the determination to win and not be beaten, is the means by which the goal of mutual improvement between fighters during combat practice is achieved. This improvement can take place in different situations for each person (34).

It is therefore important to study how combative psychological transitions are identified, given that these phenomena are not necessarily clearly demarcated, objectively or qualitatively, externally. Ultimately, it is the manner in which each practitioner responds to success, frustration, advantage, or disadvantage in their regular practice experiences that can determine their capacity to interact with their opponent effectively (34).

It thus falls upon the teacher, who oversees the teaching practice, to make ethical pedagogical decisions in instances where their students lose control. It seems reasonable to posit that such decisions can foster well-modulated environments in which hostile feelings do not lead to uncontrolled behaviour and vexatious embarrassment (bullying), both of which are closely linked to violent experiences. Similarly, it can be postulated that such occurrences are related to the abandonment of the practice. It would therefore be beneficial to be able to identify and understand the transitions between different combative experiences, as this would facilitate the modulation of practice environments that engender changes in one's perception of oneself and of those with whom one practices MA&CS (3). However, it would be beneficial to gain insight into how these authority figures manage situations when things become unruly.

This investigation presents the path thus far and is interested in finding out both the master's perception of disruptive experiences and their perception of the consequences for practitioners. This is with the understanding that, in the face of situations that specify the limits of the MA&CS combat experience, their interventions can be decisive for whether or not there is a positive development of the practitioner in the sport.

While the Integrated Technical-Tactical Model (18, 19, 38) provides a consolidated framework for developing fighting knowledge, focused on technical-tactical content, such approaches assume the fighting situation as pre-established and do not explicitly address psychological transitions toward play-fighting or brawling states. This gap, already highlighted by Barreira et al. (1), becomes particularly relevant when considering the “constraints to generate affordances” proposed in the ecological dynamics model (39).

Accordingly, the principal objective of this research, founded upon these theoretical and conceptual premises, was to identify and comprehend, through the utilisation of first-person accounts, the manner in which Martial Arts and Combat Sports instructors in Brazil perceive potential personal alterations and developments in their pupils resulting from circumstances of lack of control they encounter during combat training.

## 2 Materials and methods

The present study adopts the empirical-phenomenological approach detailed by Barreira et al. (1), which is a qualitative, exploratory and descriptive investigation. This approach is grounded in the tenets of classical phenomenology, a philosophical tradition initiated by the German philosopher and mathematician Edmund Husserl (1869–1938). It aims at apprehending the meaning of things, including those pertaining to the social and cultural dimensions of human experience. This entails a process of reflection on how phenomena present themselves to those who engage with them. In order to examine what is apprehended, it is necessary to consider both the object being perceived and the subject perceiving it. This entails analysing lived experiences, or in other words, intentionality. Instead of a strictly philosophical phenomenological consideration, carried out as a solitary analysis of lived experiences, we proceed to a phenomenological psychology that reaches out to the lived experiences of others in order to multiply the concrete experiences that make it possible to intentionally analyse the phenomenon (40–44).

The methodological procedures used are described in greater detail in the following section. These include (1) the semi-structured interview, conducted under the auspices of “suspensive listening” (42, 43), which yields an intersubjective production of experience reports, and (2) the intentional crossing, an analysis that progresses through three stages of phenomenological reduction: psychological, eidetic and intersubjective reduction (45).

### 2.1 Data collection – interviews

The central instrument of research from this perspective is, as Barreira and Coelho Jr (43). argue, the phenomenological interview, whose operative key is “suspensive listening”. The application of this approach can be defined as “data collection,” or more precisely, the intersubjective production of experience reports (45), which are then subjected to processing and analysis. This entails a semi-structured, in-depth interview, audio-recorded. The interview is conducted in accordance with a script based on the object of experience that is the subject of the research. In formulating the semi-structured script, each question is designed to avoid the use of concepts and to prompt the interviewee to refer back to an experience of their own, whether it was lived by them or witnessed directly. In other words, the question is intended to elicit information based on the interviewee's perception of events involving other individuals with whom they practised their sport. This type of formulation already adheres to the principles of “suspensive listening,” a conceptual operational device that extends the ideal of époche in interpersonal encounters (43).

As Englander and Morley [(46), p. 33] posit, the epoché enables a recognition of the intricate intertwining of the researcher's and research participants' experiences with the phenomenon under study, thereby fostering a radical non-dogmatic and open-minded approach to psychological research. Consequently, this preliminary phase necessitates a shift in perspective for the interviewer, entailing the suspension of preconceived judgments, theories, and concepts. This also engenders a transformation in the individual being interviewed, facilitating a direct engagement with their personal experiences (44). It is of paramount importance to ascertain the account of the experience as lived by the subject, rather than seeking explanations for the situation itself.

### 2.2 Participants

The participants in the study total 47 individuals from three different countries, practicing various modalities of combat sports^2^. Thus, the Brazilian participants in this research comprised 16 female masters and 20 male masters, constituting an intentional sample of 36 individuals (total *n* = 130) who provided experiential reports through interviews. The interviewees practiced a diverse range of martial arts and combat sports, including Capoeira (10), Boxing (2), Jiu-Jitsu (7), Judo (5), Karate (10), Muay Thai (6), Taekwondo (3), and Wrestling (3). – Supplementary Table S1. The Portuguese participants in this study consisted of 1 female master and 5 male masters, forming an intentional sample of 6 individuals (total *n* = 26) who provided experiential accounts through interviews. The interviewees practiced a diverse range of martial arts and combat sports, including Boxing (1), Jiu-Jitsu (1), Judo (1), Karate (2), Muay Thai (1), and Taekwondo (1). — Supplementary Table S2. The Spanish participants in this study consisted of 5 male masters (total *n* = 24) who provided experiential accounts through interviews. The interviewees practiced a diverse range of martial arts and combat sports, including Boxing (1), Jiu-Jitsu (1), Karate (1), Muay Thai (1), and Taekwondo (1). — Supplementary Table S3. It should be noted that the sample includes a considerably larger number of men than women. However, despite being one of the limitations of the present study, this low representation of women also reflects how combat sports still present disparities in inclusion regarding the female gender. This inequality is mainly grounded in historical-sociological constructions about what it means to be a woman or a man within the community (47–49)^3^.

The designation of “master” is employed here with the same flexibility as in practical contexts in Brazil. For the purposes of this research, it was sufficient for the interviewee to be recognised within their community of practice as a responsible instructor of their main sport. Consequently, the duration of the interviewees' teaching experience ranged from two to 48 years, with the minimum period of involvement in MA&CS being five years and the maximum being 54 years. In terms of age, the youngest master interviewee was 19 years old, while the oldest was 73 years old. The regions of Brazil from which the participants were drawn included the North (4), Northeast (9), Centre-West (8), Southeast (6), and South (9). Regarding the other cultures, in Portugal, the 6 participants were from the northern region of the country (Porto and Braga). In Spain, participants were primarily concentrated in the northwestern and central-northern region, particularly in Castilla y León.

In addition, those who agreed to take part in the study were informed about the purpose of the research and signed the Free and Informed Consent Form - which had previously been approved by the local Research Ethics Committee. The identities of the participants were anonymised, which is why pseudonyms were used for the interview excerpts. The criteria used to protect the identity of the interviewees is as follows: 1st letter identifying the gender of the interviewee - F or M -; 2nd letter with the initial of the teaching method; 3rd letter with the acronym of the region in which the teacher teaches; followed by the number of years the teacher has been practising the sport.

The interviews were conducted and digitally audio-recorded using a tablet or mobile phone (smartphone) between 2020 and 2023 as part of a larger project (FAPESP - 2019/11527-6) and in accordance with an extensive script. The following questions are to be considered within the scope of this research^4^ ^5^:

1. Have you ever observed your students or practitioners experiencing a loss of control during combat practices or fights?
  - Do you perceive any alterations in the manner in which the students engage in combat as a result of these experiences?
  - Do you perceive that these experiences alter the students' mode of being?

To what extent are these experiences significant?

2. Have you ever observed your students or practitioners experiencing near-misses during combat practices and fights?
  - Are you aware that these experiences result in alterations to the manner in which the students engage in combat?
  - Are you aware that these experiences result in a change in the students' way of being?

To what extent are these experiences significant?

### 2.3 Analysing

Following the completion of the interviews, the data was transcribed in full and made available in written form for subsequent analysis. In view of the considerable volume of material, there are still a number of recordings that require transcription. It must be noted that the results presented here are preliminary in relation to the entire corpus of intersubjective experience reports, as the analysis of the remaining material will continue. However, this preliminary aspect with regard to the empirical material is not reflected in the preliminary results, as the analysis adheres to the principle of saturation, allowing for the presentation of consolidated results.

From a procedural standpoint, the research can be described as a collaborative work, employing an empirical-phenomenological analysis.

The “Collaborative Work” component of the larger research project (FAPESP n° 2019/11527-6) has facilitated the simultaneous development of four studies at the Research Laboratory^6^. This approach has fostered collaboration between undergraduate and master's researchers, enabling the expeditious transcription of data and the reciprocal enhancement of analytical skills, particularly in the context of psychological reduction, as detailed below. In this research format, each researcher is involved in the process and gains a comprehensive understanding of the totality of objects involved in applying the empirical-phenomenological methodology to uncover structural or intentional aspects linked to the practice of MA&CS.

“Empirical-Phenomenological Analysis” - Once the transcriptions have been completed, the material is subjected to analysis. As it is presented in the form of a running text, the researcher is required to assume a stance of phenomenological reduction, which is comprised of three stages: psychological, eidetic and intersubjective reduction. These stages will be described in turn, although they are interrelated.

The initial stage, designated “Psychological Reduction,” entails the synthesis of the subject's experiences as conveyed in the interview. The concept is elucidated as follows: Psychological reduction is the process of reducing the original experience of the empirical subject interviewed to a singularity, thereby removing it from the natural world and describing it as an intentional awareness of the object as personally perceived. Accordingly, the objective is to consider and describe solely the perspective of the experience as lived by a single interviewee, as conveyed in their account of the interview. This entails the exclusion of content and facts that are not directly related to the object of experience. Consequently, the process of psychological reduction is conducted for each interview under examination, without any reference to the others.

In contrast, each interview is referenced in relation to the others in the process of achieving the Intentional Crossing. This is presented as a “comparative analytical process with a view to individuating the constitutive experiences of the phenomenon in question” [(45), p. 348]. Therefore, whereas in psychological reduction the set of experiences of each individual subject was of significance, the current approach is to focus on the various manifestations of the phenomenon in question, which may be described as the object of experience designated by the research objective. The cross-referencing necessitates the sustained suspension of prior knowledge, including the idiosyncrasies of the subjective-relative historicities of each interviewee's experiences, in favour of intentional equivalences between the accounts that demonstrate and reciprocally confirm facets and modes of manifestation of the phenomenon under examination. Consequently, in the context of the intentional crossing, the eidetic reduction is interpreted as an examination of the diverse manifestations of the phenomenon, based on the principles of psychological reduction. In this manner, the eidetic reduction facilitates the formation of clusters of meaning that encompass analogous experiential possibilities, as discerned through an intuitive grasp of the researcher's [(45), p. 348]. In this manner, the researcher links together the various accounts, forming the “units of meaning” of the phenomenon being analysed.

Subsequently, we arrive at the moment of greatest refinement and sophistication of investigative execution in empirical-phenomenological research, the passage to the “Intersubjective Reduction”. This is aimed at the de-implication of the intentional elements that structure the phenomenon in view and that are shuffled in natural perception. This passage reveals the “Units of Meaning” previously identified through the eidetic reduction, with the objective of identifying “reciprocal and irrevocably valid intentionalities in pre-reflexive acts that underlie the multiplicity of their singular appearances” [(45), p. 356]. The term “intersubjective reduction” is used to describe the process of identifying and making explicit the intentionality that is present in the eidetic reduction but not yet fully apparent. This intentionality is responsible for the formation of the groupings observed in the eidetic reduction, and it is this which enables researchers to perceive the common meaning of a given Unity of Meaning.

Subsequently, the material was transcribed in full and subsequently read and reread in order to identify and analyse any pre-reflective and intentional acts pertinent to the manifestation of the object of analysis.

## 3 Results

The identification of disruptive situations confirms, in the pedagogical practice reported by masters, the oscillatory dynamics described by Barreira et al. (1). A systematic examination of the expressive accounts of Brazilian, Portuguese, and Spanish martial arts and combat sports instructors, along with an investigation of their perceptions regarding situations of lack of control experienced by their students during combat practices — as well as any personal changes resulting from these experiences — allows for the identification of a series of experiential facets directly related to the object of study. The four units of meaning are as follows:

1. Combative intensification;
2. Disruptive situations;
3. Interventions by the instructor;
4. Changes in the process.

Although these results are consolidated by a saturation phase defined by the irrevocable constancy of these elements, it is important to note that these results could be further enriched by the description of a greater diversification of their manifestations, as more reports from instructors are transcribed and analysed.

### 3.1 Combative intensification

In each instance of a lack of control, the instructor identifies that combative intensification is a precursor to the situation. It is noteworthy that for these individuals, this intensification is not perceived as alien to the struggle or even as undesirable. The reports indicate that the combative challenge necessitates this intensification, thereby suggesting that there is an implicit understanding on their part that the conditions of the practice environment should facilitate the presence of adequate tension. “I believe it is beneficial for individuals to experience situations that require them to persevere and overcome challenges independently. I believe this is a necessary requirement” (F.K.S.26), as well as the account of a participant from Spain who stated: “As a teacher, I don't like to interrupt them and say, ‘Hey, that's not okay,’ no. I prefer that people adapt to whoever is in front of them” (M.K.ESP.35). The question thus arises as to how one might effectively guide individuals along this trajectory. Nevertheless, augmenting the challenge through combative intensification also entails an elevated risk of losing control, a scenario that those who practise must be prepared to face. As M.K.S.50 aptly observed, “everywhere there will be someone wanting to test you.” As evidenced in the excerpt below, the term “provoking lack of control,” as defined by a master, does not imply a desire for absolute lack of control. Instead, it entails the expansion of the combative challenge and the modification of combat to create a situation in which the practitioner is compelled to maintain control despite adversity.

“So, in karate I provoke lack of control, I take away the students’ balanced environment (..) because there are children who don't lose control. Then they don't necessarily need to be out of control.” (M.K.N.33)

Given the expectation of intensifying the combat, it is imperative not to avoid confrontation. Despite the absence of evidence indicating a lack of control in the absence of prior tension, it is crucial to recognize the potential for combative intensification and to address it accordingly.

“He was very confident, and I said, ‘You have to manage yourself because there are more rounds and you’re going to get tired.’ And in reality, he got so exhausted that he started losing—he loses because he gets hit—and then he looked at me as if to say, ‘Get me out of here, end the fight.’ I said, ‘No! Now you're going to stay because that was the decision you made. You chose not to listen.’ And he had to keep taking hits for the rest of the fight.” (M.M.PT.15)

Furthermore, it is evident that the individual responsible for overseeing the class must possess a keen understanding of the nuances involved in differentiating between the appropriate tense and the incorrect tense, on a case-by-case basis, in the context of combat. As one capoeira instructor observed:

“(..) normally [these moments of lack of control] refer to the mestre or mestra of the roda; the one who is there coordinating that roda, the one who needs to be much more attentive than the others. And that's the one who's going to set the energy there with what he sings, with what he instigates (..) [What] is he instigating for the game to happen? Something firmer?” (F.C.S.30)

With this in mind, the same instructor also argued that to prepare her students for these moments, “in training (..) you always need to be working on this [which] is ‘the hot foot but the cold head’” (F.C.S.30).

### 3.2 Disruptive situations

In most reports of uncontrolled actions, a feeling of hostility towards the opponent predominates. Not only it is necessary to point out that the motivation for this feeling varies significantly, depending on the context and what is going on between those involved - including, sometimes, rivalries prior to the situation witnessed - but, above all, to emphasise that, despite being predominant in the reports, lack of control is not essentially linked to hostility. There are discontrols perceived as technical and motor, when, for example, practices are proposed in which the blows should not hit the opponent and they do, or when the force applied exceeds the expectations of the practice. In this type of physical intensification, it is not uncommon for these uncontrolled actions not to be noticed by the person firing them until they hurt the other or trigger disruptive situations based on the opponent's reactions when they also intensify their blows until, for example, hostility prevails between them. However, there are situations in which even because the opponent is unable to react, the lack of control becomes evident as disproportion and inattention to what is happening to the person being hit. One such episode is well illustrated in this extract:

“I was teaching and it was kumite, and he started hitting her in the uchi komi exercises - on the counterstrike, and you can't hit on the counterstrike. So he kept hitting her, until there came a point when we got to a freer exercise, and he literally hit her (..) and he was a green belt and she was a white belt.” (F.K.S.26)

Conversely, when a practitioner, cognizant of their inferiority in a competitive context, encounters an experienced opponent who exhibits a lack of attention and disproportionate dominance, that is, exerting control without consideration for their physical or technical limitations – which may include factors such as size, strength, and rank within the sport – the resulting reaction may manifest as an uncontrolled hostile response.

“One of them was stronger than the other, he gave him a crushing blow. The other felt in an uncomfortable situation (..) and wanted to get out of it (..) by kicking him: he kicked the boy a couple of times; that could be characterised as a kick or he thought it wouldn't be [understood as an unauthorised blow in Judo]. And the other guy didn't like the situation and kicked back [unauthorised]” (M.J.CO.42).

While factual markers such as belt or weight can be important in demarcating expectations between opponents, they are not always the most decisive factors. Notwithstanding the similarities in these and other domains, the imposition of an unanticipated or unacceptable dominance can be associated with a state of lack of control.

“And there came a moment when this guy (..), he got very angry that the other guy wasn't accepting him dominating the fight. And he went into a sequence (..), he came out like this, you could see in his eyes that he wasn't thinking about what he was doing. He went all out, he pushed the guy into the wall, he grabbed the guy and pinned him against the wall with both hands.” (F.K.S.26)

A Portuguese karate instructor summarizes some of his perceptions regarding his students' disruptive situations as follows:

“Sometimes they are doing an exercise and it starts to become evident that the person is showing behavioral changes, that they are no longer even thinking about what they are doing, that things start to go wrong, and that the person becomes very anxious. And they might start to clash with the other [person] or, very often, what happens is that, due to physical or technical inferiority, we know that even the person who is becoming more uncontrolled won't resort to violence because they know they’re not capable. But perhaps it will reach a point where they either start crying, storm out saying they won't continue, or something along those lines.” (M.K.PT.42)

### 3.3 Interventions by the instructor

In the reports, as soon as the instructor identifies a shift in the nature of the combative sports phenomenon towards a violent one, from one characterised by competitive aggression to one involving physical violence, they intervene. The intensity and manner in which the interventions are presented vary significantly depending on the circumstances of each situation. In extreme cases, it may be necessary to physically separate the parties involved and request that they leave the classroom. However, when a student attacked him excessively during a capoeira game, the interviewed mestre's reaction exemplifies the typical approach to intervening directly in situations where control is severely compromised. The objective is to respond definitively in combat, thereby underscoring the implications of a lack of control in the context of the fight.

“And then [when] he went to play with me, (..) [he] fell in with violent and quick blows (..). And at that point I got out, ducked; when I ducked he was facing me, I headbutted him, he took five steps backwards, when he got to his feet, I hit him with a jumping kick and threw him into the wall.” (M.C.S.25)

A Spanish Jiu-Jitsu instructor also describes how he carries out a combative intervention with some beginner students who tend to strike harder when they first arrive:

“Some come in — and it's normal — hitting a bit harder, so I respond with a light strike. I almost never hit very hard here, I just go for the liver. I strike the liver hard, they fall to the ground, then recover a bit. And they become a bit more aware.” (M.MMA.Jiu.ESP.19)

Similarly, intervention can be initiated by other practitioners. This was demonstrated when an instructor sought assistance from a colleague of a similar rank to rectify an incident in which the practitioner was striking a novice with excessive force:

“He went to get some water and I called another of his green belt colleagues, a little younger, and I said ‘hit him!’ He said ‘what?’; I said ‘hit him, he's hitting her’ (..). He (..) squeezed the guy. (..) The other black belts were already realising what was happening: no one laid a finger on him, but everyone squeezed. The guy paid for his sins in class. He didn't have to say anything else” (F.K.S.26).

In addition to explicitly confrontational interventions, there are also cases in which the approach takes the form of conversational guidance directed at the student who has lost control—or, in cases where the student is a child, at their family.

“There's an athlete who was losing the first match; it was going very badly. And after the first round ended, she went to rest. During the break, I said: ‘Breathe. Open your eyes, look at what's happening. There are still two rounds left—you can win. That was just the first one. The match isn't going as badly as you think.’” (M.M.PT.15)

Furthermore, in the dialogues under discussion, the close relationship between the athlete's emotional state in combat and the martial arts and combat sports (MA&CS) technique employed is particularly noteworthy.

“And then you have to work on that emotion ‘no, wait, whose fault is that? Whose fault is it, who hit or who got hit?’ In the case of karate, ‘did you know how to defend? You knew the blow, so it's your fault that you didn't defend. What are we going to do about it? Are we going to make it right? Are we going to be quicker? Are we going to be more attentive? Let's connect with each other to find out?’” (M.K.S.40)

There are situations in which a warning has been imposed and only then has dialogue taken place:

“So I got up and went over there and said ‘what's going on with you? Let's calm down’ (..) ‘Calm down, this isn't the time and I'll talk to you later’. And I did. When we came here, then I called him, we went to talk, me, him and the other teacher who taught him.” (F.T.N.35)

However, what are the results of the master's interventions?

### 3.4 Changes in the process

“It's better that he pops up here now, at the age of ten, (..) I know what his limit is and we work in here, than when he's eighteen and shoots someone, when he's twenty and takes the car and runs over someone. I want it here!” (M.K.N.33)

The comparison of this narrative illustrates the two contrasting perspectives on the intentional crossing as a process of development from the perspective of the instructors. The initial hypothesis posits a tragic scenario wherein an individual's capacity for self-control is compromised in adulthood, leading to a lack of control within the social structure. The second example, situated within the context of the master's practice of combat and guidance, is the episode of a fight between children that escalated into a brawl. At both extremes, the crux of the matter is the complete rupture of personal boundaries, that is to say, disruptive experiences that are consummated in violence. The process of going to the limit at MA&CS, whether extreme or not, enables the resolution of disruptive experiences through learning and development. This is achieved by addressing the increased tension in a constructive manner, preventing its breakdown.

The disruptive situations reported indicate the existence of distinct spheres of transgression and transformation in the practitioner. In a more technical context, where the limit in question pertains to motor gestures and their execution, the guidance provided by the teachers tends to focus the students' attention and efforts on the aspects of their actions that they can control.

“Yes, I mean, when they compete, they realize that if they act that way, they will get hurt. That is, if they attempt a heavy-handed strike and cause damage without paying attention to technique—without trying to be fast, leaving themselves wide open and dismantling their guard, throwing wild punches—they realize they will get hurt, because they are hit hard in competition. (..) But then, in competition, the experience usually confirms that I was right about what I had been saying. So they come back to training and say, ‘It's true, you were right.’ And we go back to square one. And we start over with something else. But yes, when things get a bit out of control and they are struck hard, they really don't like it. They say, ‘I need to avoid that situation’.” (M.B.ESP.12)

It is crucial to highlight that, as illustrated in the aforementioned account, fighting entails a distinct yet intimate interplay between the technical motor sphere and the relationship with the opponent. In this intercorporal relationship, the exclusive focus on the execution of technical combative skills has significant implications.

“You have to see if your karate has helped to centre you or alienate you. You're going to hit everyone: then it hasn't helped you, it's alienated you. (..) [Being centred] is trying to look after your own, right; and you have to see the other person too.” (M.K.S.50)

The aforementioned alienation is exemplified by the account of a practitioner who engaged in combative behaviour while simultaneously exhibiting a lack of consideration for others. This illustrates that unbridled impetuosity is not synonymous with the attention or capacity required to maintain one's own well-being. In a symptomatic response, the student in question ceased participation in the practice when he was confronted with the inversion of the physical advantage. “Upon realising that it was relatively simple to overcome the smaller opponent, but considerably more challenging to prevail over the larger one, he ultimately chose to abandon the practice” (M.K.S.40). Conversely, following the intervention of the instructor, whereby more seasoned practitioners were encouraged to apply pressure, the student who had struck the novice exhibited a notable shift in attitude. This was attributed to the fact that, prior to this intervention, no individual had adopted a more confrontational stance towards him. He believed that he could exploit others and, as a consequence, he would initially target the most vulnerable, who were also the least experienced. In accordance with the initial account of this Sense Unit, the masters perceive a broader scope for this transformation in the individual's life. They posit that “on occasion, within the context of training, these minor disagreements can facilitate the development of a more measured response, preventing the individual from externalising their frustrations and potentially compromising their composure in situations that may be disadvantageous to them outside of judo practice” (M.J.CO.42). This allows us to gain a deeper insight into the influence that exposure to potentially disruptive experiences can have on a practitioner's development.

As illustrated by the account of a Portuguese karate instructor:

“If I can keep them from giving up because of it, change happens. It happens through that. Of course, it is also linked to the student's own growth—physically and technically—but I believe that (..) if the student remains over time, they are able to contextualize those experiences and understand them. To realize that, at the time, ‘my technique was lacking, the other had more skill, it was very difficult to succeed, but now I’m on a more equal footing.’” (M.K.PT.42)

The condition for change, however, is related to the realisation of what has occurred. On certain occasions, this requires more than merely adjusting the motor gesture to align with the intentions and intensity appropriate to the struggle and its consequences. This occurs in the dynamics of the succession of experiences themselves, as well as involving their joint evaluation. “It undergoes significant alterations.” The individual may either come to recognise that the lack of control is a factor that necessitates a greater degree of attentiveness and thus effect a change, or alternatively, they may abandon the practice entirely (M.T.CO.33). Therefore, the disparate accounts of the cessation of AM&EC illustrate that there is no assurance that the disruptive experience will inevitably result in growth and development. On occasion, change did occur. On occasion, however, the student in question relinquished their engagement with the art practice, citing an inability to continue. (M.K.S.40) In order for change to occur, the instructor emphasise the necessity of appropriate intervention guidance:

“It's not always without guidance. (..) I think it's important for students (..) to have guidance on this. And from the moment you manage to affect these people, in the sense of showing what their reading of what happened is, (..) then I think we can bring about some kind of change.” (F.C.S.19)

But, as she also emphasised, “of course, for this to happen (..), [the student] needs to be willing, they need to be open to this possibility. You're never going to teach someone who thinks they have nothing to learn” (M.T.C.33). Disruptive situations in which the triggering issue is not technical, but involves escalating combative intensity until, at the limit, it culminates in rivalry between opponents, seem to generate more unwillingness to learn. Analysing by the instructor' reports, situations like this are more difficult for the students to recognise their fault or how their personal position was a determining factor in the lack of control. However, as F.C.S.19 said above, when the intervention leads the student to realise, be affected by and read the situation, change happens. These are mostly subsequent assessments in which, with more emotional distance, reflection on what happened and its consequences favours a move towards self-control:

“He was able to realise that it was actually a team competition and it ended up hurting the whole team. So afterwards he said ‘well, I could have held back a bit’; so I think yes, it was a learning experience.” (M.J.CO.42)

The process of becoming aware of other implications and damages related to the loss of control is an essential component of an endeavour for self-control. This awareness extends beyond the individual to encompass the other people affected and the wider community of practitioners within which one is inserted. It also encompasses reflective processes that facilitate the commitment and accountability of the person with their MA&CS modality.

“Yes, I think there's (..) a bit of compromise there (..). And I think that depends on what's dear to each of those people. So, (..) when a situation arises and that person receives guidance from the situation that happened, whether it's a conversation, a game that shows them what happened, I think they start to realise themselves more and start to ask themselves (..): ‘what else is there?’ And I think that asking ‘what else is there?’ generates some reactions of taking responsibility for oneself and taking responsibility for others, which I think automatically starts to extend into the person's life.” (F.C.S.) (F.C.S.19)

It is also evident that changes following disruptive moments play a significant role in shaping one's perception of self and personal limits. When asked about the importance of such experiences, two different instructors responded:

Interviewer: What is the importance of these experiences?

Instructor: “Exactly that — to see reality, to perceive reality.” — (M.Jiu.PT.12)

Interviewer: What is the importance of these experiences?

Instructor: “Because it's important that they go through this phase. So they can be close to reality. Otherwise, if they are not close to reality, they will never reach that psychological level. Do you understand?” (M.K.PT.44)

Interviewer: “What psychological level is that?”

Instructor: “The truth. The truth (..) about your loss of control. In order to be able to control yourself, you have to lose control! How do you lose control? You can't pretend to be out of control. You have to actually be out of control. How do you reach that state? Only by provoking the situation. How do you provoke it? You create situations in which students lose control with one another. That's how they evolve. And how is that done? It's the coach who has to make it happen. (..) The coach orchestrates all of these situations. (..) And then there comes a time when the student is so used to it, so accustomed to these moments, that eventually they stop losing control. They’ve adapted to the stress. They no longer lose control in that context. They are now able to remain composed under stress. Do you understand?” (M.K.PT.44)

### 3.5 Synthesis of results' analysis

The potential for disruptive situations, as well as for instructor interventions, is vast, as is the scope for change in students. However, through intentional analysis, this variety can be reduced to a process of change. This process commences with the attainment of technical and motor proficiency, with the objective of preventing uncontrolled behaviour. Such occurrences are more prevalent among novices who have yet to incorporate the motor gestures of the sport and lack the capacity to regulate the force of their strikes. At this stage of the process, there is an overlap between the increase in momentum and the imposition of actions, which may be either voluntary or involuntary, and the actions of the opponent. This requires greater attention and consideration for those being trained and fought with. A more expansive sphere, which incorporates the preceding ones, is reflexive and pertains to the self-responsibility associated with the ramifications of the lack of control experienced. This is not merely conceptualised as control, but as self-control.

It is therefore an experiential process in which, gradually, the consequences of the motor actions correlated to the disruptive episodes, in different domains, lead to the changes that characterise the practitioner's development. When motor gestures go beyond the limits of practice and, in the extreme, hurt, the sphere in question is bodily and empathetic, relating to technical motor control and lack of control, as well as attention to the other. On the other hand, when the emotions between the opponents escalate wildly in their actions and reactions, we enter the psychic sphere of the disruptive experience. Finally, the psychosocial sphere stands out when actions are driven by a sense of self-affirmation that transgresses the norms and the field of what is acceptable in combat practice. This differentiation of spheres is relevant because, although they can manifest themselves intertwined in the disruptive situation, a correct apprehension of the domain that determines the loss of control allows for appropriate intervention and guidance on a case-by-case basis. When these experiences and consequences are tasted - or witnessed - and are appropriated in their connection, there is, in the first instance, a change in perception. By extending to previously unapprehended consequences, this perception directs action not only to avoid deviations from violence, but to lead to the success of the practice. The efficiency that is gained for the success of the practice doesn't always mean victory in the fight, since in certain circumstances, such as those where there is a clear advantage over the opponent, the success of the practice lies more in allowing a challenging fight than in imposing oneself and winning promptly. This whole process only leads to development by virtue of a focus on the best consequences of the actions, be they bodily, psychological or psychosocial, consequences that actualise the expectations and values of the MA&CS practised. Without the adequate interventional participation of the instructors, the actions would have random and arbitrary consequences, much more susceptible to violence which, because of its proximity, is a threat to the ability to fight.

## 4 Discussion

The definition of a fight as “a bodily confrontation in which one opponent tries to physically overcome the other” [(28), p. 422] suggests that the act of fighting may give rise to ambiguous feelings among those involved, as well as among those who observe the fight (51). As Barreira and Telles (52) observe, this ambiguity is intrinsic to the practice of sport. While the opponent is both desired and required in the context of competition, they also present an obstacle to the desired outcome.

The results presented here not only dialogue with the phenomenology of combat proposed by Barreira et al. (1), but also confirm its applicability in the pedagogical field. Moreover, they highlight that methodological and conceptual gaps present in models such as the Integrated Technical-Tactical Model (38), and the ecological dynamics model (39), may be overcome by incorporating pre-reflective psychological dimensions identified in the present study, including those related to affective and ethical affordances modulated in the analyzed pedagogical situations. Our data indicate that in real pedagogical situations, instructors' interventions during episodes of emotional disruption not only restore motor order but also modulate affective and ethical affordances, expanding the scope of existing models beyond mere technical-tactical organization.

This presence of the other in combat, which is alive and constant, is what is taken into account to incite and modulate movement in the fight (52), since the perception of the presence of the other accompanies the “impossibility of full control of our actions” [(52), p. 97]. As has also been noted (32), in MA&CS, unpredictability is a characteristic of the hand-to-hand experience and, due to the act of restricting bodily mobility - the purpose of the fight - there is a bodily constraint that “tends to correlate with a moral constraint (..)” [(29), p. 432]. Therefore, “The subject of the physical struggle finds in the antagonism of the opponent the adversity that proposes and intensifies the challenge for self-improvement, a figure of ethical life.” [(29), p. 442].

If one were to interpret this phenomenon through the lenses of physicalism or idealism, it would be impossible to discern the duality of feelings that is intrinsic to situations where the scene takes place in the experiential domain of the body-to-body, such as in the case of physical altercations. It is therefore not uncommon for the perspective of a third-party observer of a physical altercation to perceive the event as violent, due to a misapprehension of the experience of a hand-to-hand dispute (31). From the external, third-person perspective, the most intense and incisive fights appear to contradict any possibility of empathy between the combatants. Without attention to the specificity of the interpersonal tension and mutual improvement evident in these fights (53), there would be no process of recognising the other as other between the practitioners. In contrast, a phenomenological analysis indicates that “the practice of fighting entails particular attention to the opponent, rather than, as the notion of confrontation might suggest, their elimination” [(29), p. 416].

The results demonstrate that MA&CS instructors perceive potential shifts in their students' behaviour due to the fact that disruptive situations are ethically and pedagogically based on the objective that the learner, in a borderline moment, should not lose control and, if they do, that they should gain from the experience. This underscores the significance of comprehending how the individuals involved in the action perceive and experience the moments in question. The improvement of self-control is demonstrated as an ongoing ethical imperative within the context of the fights, with the objective of achieving a personal transformation that simultaneously entails the challenge of self-control and the domination of the opponent [(29), p. 440]. In light of these findings, it can be concluded that the ethical dimension of MA&CS practice is primarily shaped by the relationship between the apprentice and the master (17, 28).

It is important to acknowledge that solely presenting the student's proposed modifications based on the instructor's input may be a potential limitation, as it fails to consider the learners' perspectives. However, due to the difficulty of accessing such situations of lack of control, as well as the confidentiality required by the ICFs, this contact could not be established during the course of the research.

## 5 Conclusion

This study complements and expands the theoretical contributions of Barreira et al. (1), by demonstrating how MA&CS masters identify and intervene in combative transition processes. Therefore, with the aim of identifying and understanding, through first-person experience reports, how Martial Arts and Combat Sports teachers perceive possible changes and personal developments in their students caused by disruptive situations experienced by the latter in combat practices. This perception of changes resulting from a disruptive situation is supported by the combative intensification that is essential in combat practices, as well as by the appropriate intervention needed when control is lost.

It can therefore be concluded that, in the face of disruptive situations, the interventions of masters aim to modify the practitioner's perception towards greater physical, technical and emotional self-mastery. It can be inferred that the psychological transition between corporal fighting and violence not only occupies an ethically central place in the practice of AM&EC, but that the proper management of those who teach and those who learn a fight is decisive for a positive change in the student, i.e., their development.

### 5.1 Practical applications

Regarding the potential practical applications of this study, it is first necessary to exercise notable sensitivity in order not to interpret the data as a “ready-made recipe” for MA&CS instructors to use in disruptive situations with their students. This is particularly relevant, as each situation and each learner will require specific considerations. That said, this research aims to contribute to practice in moments when instructors perceive that the fight no longer seeks mutual improvement among participants and that hostility is heightened. Interventions can then be carried out, which, depending on the student and the moment, may involve dialogue, a combat exercise, or a punishment by withholding the opportunity to continue the fight.

Beyond the immediate management of disruptive episodes, it is important to highlight that such moments can also be transformed into opportunities for learning and personal growth. Instructors may foster resilience, emotional modulation, and the ability to sustain combative engagement without hostility, guiding practitioners towards constructive outcomes. In this sense, rather than being reduced to a disciplinary measure, the intervention can open a path for the practitioner to appropriate the disruptive experience as a moment of improvement, contributing not only to technical and ethical development but also to overall well-being.

### 5.2 Limitations

However, it should also be pointed out that observing the personal development of Martial Art and Combat Sport practitioners after a disruptive situation only from the teacher's perspective appears to be the greatest limitation of this research. This is because it does not cover a possible perception of self-development resulting from a situation that goes beyond the limits of what is acceptable in a hand-to-hand dispute, nor does it contemplate the way in which this first-person experience could occur. In this way, a follow-up to this piece of research could be to find out how, from a first-person perspective, their own disruptive experiences and possible changes and personal development occur in those teachers who perceive that there can be a process of change in their students with their interventions.

Regarding the study's limitations, it should be noted that the low number of female participants in the sample limits the generalizability of the results, as approximately 64% of the sample are men, while only 34% are women. Therefore, there is a need for future studies with greater equity between the number of women and men in the sample.

## Data Availability

The raw data supporting the conclusions of this article will be made available by the authors, without undue reservation.

## Footnotes

^1^For the purposes of this work, "disruptive" is taken to mean training or competition situations that present a psychological tension that puts pressure on the fight against violence, risking distorting the "fight" phenomenon into a "brawl" phenomenon. Different psychological states are related to this tension that characterizes the situation as hostile, including anger and fear. However, it is necessary to know to what extent these states determine the combative phenomenon, that is, make it a fight or a brawl, which involves considering the relationship between the practitioners.

^2^This study did not aim to compare levels of development or institutional recognition of martial arts and combat sports across countries. Participants were not selected according to authority, representativeness, or competitive level, but through local contacts, teacher referrals, and availability to be interviewed. Consequently, their institutional, recreational, or competitive insertion is random. This procedure is consistent with the phenomenological approach, which privileges the analysis of lived experience rather than national or institutional profiling.

^3^It is also relevant to point out that this research topic (the participation of women in MA&CS) is a subject of interest for the research group [50].

^4^The interviews were conducted in the participants’ native languages. Accordingly, Portuguese was used in Brazil and Portugal — with distinctions between Brazilian and European Portuguese — and Spanish was used in Spain.

^5^It should be noted that different researchers conducted the interviews. In Brazil, interviews were conducted by Cristiano R. A. Barreira; in Portugal, by Maria Gabriela dos Santos; and in Spain, by Yan Lázaro dos Santos. The latter two researchers were also responsible for a large part of the transcription of the Brazilian interviews and, prior to the fieldwork abroad, were specifically prepared to conduct interviews under the mode of suspensive listening, becoming familiar with both the operative procedures and the semi-structured script. Nevertheless, methodological rigor was evident in the analyses and was sustained through the collaborative work carried out by the research group.

^6^The Phenomenology and Body Practices Research Group (GFEPAC) holds weekly meetings at the University of São Paulo's Ribeirão Preto School of Physical Education and Sports (EEFERP-USP).
