# Supplementary material for: Navigating disruptive experiences in combat sports: perspectives of Brazilian, Portuguese, and Spanish masters on emotional control, resilience, and well-being
Source: Front Sports Act Living. 2025 Oct 23;7:1675930. doi: 10.3389/fspor.2025.1675930 (PMC12588995; doi:10.3389/fspor.2025.1675930)
Supplement: Supplementary file 1 [file Datasheet1.pdf]

Supplementary Material

1 Supplementary Tables

Table 1: data from each person in Brazil who gave an interview already analyzed for this section.

Pseudonym	Age (years)	Gender	Modality(ies)	Practical experience (years)	Teaching experience (years)	Region of Brazil
1 - F.K.S.26	30	female	Karate	26	9	South
2 - M.K.S.40	53	masculine	Karate	40	+20	South
3 - M.T.CO.33	47	masculine	taekwondo	33	+20	centre-west
4 - F.J.CO.28	44	female	judo/jiu jitsu	28/25	-	centre-west
5 - F.C.SE.19	36	female	capoeira/karate	19/17	-	southeast
6 - M.K.S.50	60	masculine	Karate	50	40	South
7 - F.K.NE.21	26	female	Karate	21	-	northeast
8 - M.K.CO.32	42	masculine	Karate	32	-	centre-west
9 - M.K.SE.47	64	masculine	Karate	47	-	southeast
10 - M.W.CO.12	27	masculine	wrestling/jiu jitsu	4/12	2	centre-west

11 - M.M.SE.12	28	masculine	muay thai/jiu jitsu	12/10	-	southeast
12 - M.K.NE.33	44	masculine	Karate	33	-	northeast
13 - F.M.SE.5	28	female	muay thai	5	-	southeast
14 - M.J.CO.42	46	masculine	judo/jiu jitsu	42/30	29	centre-west
15 - F.T.N.35	73	female	taekwondo	35	-	north
16 - M.B.N.24	38	masculine	Boxing	24	17	north
17 - M.C.CO.25	50	masculine	capoeira	25	-	centre-west
18 - F.C.S.30	50	female	capoeira	30	23	south
19 - F.T.SE.12	22	female	taekwondo	12	-	southeast
20 - M.Jiu.NE.20	34	masculine	jiu jitsu	20	-	northeast
21 - M.K.NE.39	53	masculine	Karate	39	-	northeast
22 - M.C.N.30	43	masculine	Capoeira	30	25	north
23 - M.C.S.54	67	masculine	Capoeira	54	48	south
24 - F.Jiu.S.12	39	female	jiu jitsu/ wrestling/ muay thai	12/ 11/ 8	5	south

25 - M.C.NE.37	51	masculine	Capoeira	37	-	northeast
26 - M.J.S.51	63	masculine	Judo	51	40	south
27 - M.M.S.24	40	masculine	muay thai	24	19	south
28 - F.C.SE.25	37	female	capoeira	25	-	southeast
29 - F.C.NE.26	45	female	capoeira	26	-	northeast
30 - F.M.NE.11_31	31	female	muay thai	11	-	northeast
31 - F.M.NE.11_27	27	female	muay thai	11	-	northeast
32 - F.K.NE.17	19	female	Karate	17	-	northeast
33 - M.W.N.12	28	masculine	judo/ jiu jitsu/ wrestling	12/ 12/ 4	5	north
34 - F.C.S.20	34	female	capoeira	20	15	south
35 - M.J.CO.15	22	masculine	Judo	15	-	centre-west
36 - F.C.CO.21	43	female	capoeira	21	-	centre-west

Table 2: data from each person in Portugal who gave an interview already analyzed for this section.

Pseudonym	Age (years)	Gender	Modality(ies)	Practical experience (years)	Teaching experience (years)	Region of Portugal
1 - M.M.PT.15	39	masculine	Boxe / muay thai	10/15	-	north
2 - M.T.PT.50	70	masculine	taekwondo	50	+40	north
3 - F.J.PT.33	36	female	Judo	33	17	north
4 - M.Jiu.PT.12	39	masculine	jiu jitsu	12	6	north
5 - M.K.PT.42	58	masculine	Karate	42	37	north
6 – M.K.PT.44	62	masculine	Karate	44	44	north

Table 3: data from each person from Spain who gave an interview already analyzed for this section.

Pseudonym	Age (years)	Gender	Modality(ies)	Practical experience (years)	Teaching experience (years)	Region of Spain
1 - M.K.ESP.35	40	Masculine	karate	35	-	northwest/central-north
2 - M.MMA.Jiu.ESP.19	47	Masculine	MMA/jiu jitsu	19	-	northwest/central-north
3 - M.B.ESP.12	26	Masculine	Boxe	12	-	northwest/central-north
4 - M.M.ESP.22	42	Masculine	muay thai	22	-	northwest/central-north
5 - M.T.ESP.31	45	Masculine	Taekwondo	31	-	northwest/central-north
